# Oral Administration of Compound Probiotics Improved Canine Feed Intake, Weight Gain, Immunity and Intestinal Microbiota

**DOI:** 10.3389/fimmu.2019.00666

**Published:** 2019-04-02

**Authors:** Haiyan Xu, Weiqiang Huang, Qiangchuan Hou, Lai-Yu Kwok, Wuri Laga, Yanjie Wang, Huimin Ma, Zhihong Sun, Heping Zhang

**Affiliations:** ^1^Key Laboratory of Dairy Biotechnology and Engineering, Ministry of Education, Inner Mongolia Agricultural University, Hohhot, China; ^2^Key Laboratory of Dairy Products Processing, Ministry of Agriculture and Rural Affairs, Inner Mongolia Agricultural University, Hohhot, China

**Keywords:** probiotics, intestinal microbiota, immunity, canine, health

## Abstract

Probiotics have been used successfully to promote human and animal health, but only limited studies have focused on using probiotics to improve the health of hosts of different age. Canine microbiome studies may be predictive of results in humans because of the high structural and functional similarity between dog and human microbiomes. A total of 90 dogs were divided into three groups based on dog age (elderly group, *n* = 30; young group, *n* = 24; and training group, *n* = 36). Each group was subdivided into two subgroups, with and without receiving daily probiotic feed additive. The probiotic feed additive contained three different bacterial strains, namely *Lactobacillus casei* Zhang, *Lactobacillus plantarum* P-8, and *Bifdobacterium animalis* subsp. *lactis* V9. Serum and fecal samples were collected and analyzed at four different time points, i.e., days 0, 30, and 60 of probiotic treatment, and 15 days after ceasing probiotic treatment. The results demonstrated that probiotics significantly promoted the average daily feed intake of the elderly dogs (*P* < 0.01) and the average daily weight gain of all dogs (*P* < 0.05), enhanced the level of serum IgG (*P* < 0.001), IFN-α (*P* < 0.05), and fecal SIgA (*P* < 0.001), while reduced the TNF-α (*P* < 0.05). Additionally, probiotics could change the gut microbial structure of elderly dogs and significantly increased beneficial bacteria (including some *Lactobacillus* species and *Faecalibacterium prausnitzii*) and decreased potentially harmful bacteria (including *Escherichia coli* and *Sutterella stercoricanisin*), and the elderly dogs showed the strongest response to the probiotics; the relative abundance of some of these species correlated with certain immune factors and physiological parameters, suggesting that the probiotic treatment improved the host health and enhanced the host immunity by stimulating antibody and cytokine secretion through regulating canine gut microbiota. Furthermore, the gut microbiota of the elderly dogs shifted toward a younger-like composition at day 60 of probiotic treatment. Our findings suggested that the probiotic treatment effects on canine health and immunity were age-related and have provided interesting insights into future development of probiotic-based strategies to improve animal and human health.

## Introduction

Probiotic are defined as “live microorganisms, which confer health effects to the host if administrated in sufficient amounts” by FAO/WHO (1). Probiotics have shown favorable effects on the host's health including production of antimicrobial substances to inhibit the colonization of pathogenic microbes (2), regulation of host immunity and metabolism, and improvement of intestinal barrier function (3). Additionally, probiotics can attenuate major age-related changes in microbiota composition, and it has been shown to be able to promote longevity in mice via suppression of inflammatory processes in the colon (4). Most existing experimental data manifests that probiotic use is safe for most populations, but not all probiotics are effective for all disease, hence, the safety and function of each probiotic should be rigorously evaluated. Herein, the beneficial effects of probiotics are achieved through modulating the host intestinal microbiota composition (5).

A number of studies have confirmed that the gut microbiota plays a pivotal role in maintaining health of host, whether human or animals. The gut bacteria were demonstrated to influence nutrition intake, energy expenditure, physiological, and metabolic functions of the host and drive the immune response, thereby contributing to the host's health status (6). Nevertheless, many factors can affect the composition of gut microbiota such as diet, age, disease and gene. Among these factors, aging is often associated with gut dysbiosis that is characterized with the reduction of beneficial microbes. This in turn causes negative health impacts like increase in leakiness of the intestinal barrier, excessive secretion of proinflammatory cytokines, and chronic inflammation (7). Probiotics can regulate local inflammation by modulating the production of cytokines like TNF-α and IL-6, then enhance host immunity (8). Moreover, the intestinal microbiota are confined by a multilayered system that is consisted of both physical and chemical barriers together with the innate and adaptive immune systems (9). Herein, the secretory IgA (SIgA) is the dominant antibody type found in mucosal secretions, which could regulate the intestinal microbiota and its development from birth to adulthood (10), and probiotics such as some *Lactobacillus* can promote the secretion of SIgA to form a protective layer between the mucosa and microbes in host, thus inhibiting pathogen growth (11).

Canines are considered animal models for human microbiome research because of the high structural and functional similarity between dog and human microbiomes (12). Dog microbiome studies may be predictive of results in humans. Thus, dog studies provide a double benefit: for dogs directly and for the potential to generalize to humans. Although the beneficial effects of probiotics have been extensively researched in humans and animals (13), the precise mechanisms of probiotic-based immune modulation is not entirely clear, as well as the personalized gut microbiota leads to the difference in the effect of probiotics on the host, and the difference was larger in different age individuals, hence the effect of probiotic on different age host is distinct, whereas it was paid limited attention. In addition, it has become a more common practice to supplement with probiotics, but the efficacy of probiotic application greatly varies with low predictability, whose reason is not entirely clear.

A probiotic mixture was used in the present study. It was a high-dose viable lyophilized bacterium that contained three well-characterized probiotic strains, namely *Lactobacillus casei* Zhang, *Lactobacillus plantarum* P-8, and *Bifidobacterium animalis* subsp. *lactis* V9. *Lactobacillus casei* Zhang could upregulate the expression of CD4, CD8, and CD27 in whole blood and alleviate abnormality of red blood cells (14), suppress the level of serum TNF-α while enhance the level of SIgA (15). *Lactobacillus plantarum* P-8 promoted weight gain and feed intake, as well as elevated the level of SIgA and IgG in broiler chicken (16). The species *Bifidobacterium animalis* subsp. *lactis* V9 has been shown to improve and maintain the human gut microbial homeostasis (17). The Pacific Biosciences (PacBio) single-molecule, real-time sequencing technology (SMRT) is a promising third-generation high-throughput technique that is based on DNA polymerization (18). This powerful technology can generate long sequence reads and thus bacterial profiles at the species level. It has been used for describing the microbiota composition across a wide range of environmental samples (19, 20). For example, our laboratory has successfully investigated the bacterial diversity in infant formula (21) and human fecal samples using the PacBio SMRT platform (22).

In the current work, we evaluated the effects of a multi-strain probiotic compound on canine health through examining some basal physiological parameters (namely respiration rate, breathing rate, body temperature, feed intake, and weight gain), and the immune function, and monitoring the changes in the intestinal microbiota of the canine subjects before, during, and after continuous probiotic supplementation. We also aimed to explore the potential mechanisms of canine immune modulation by the probiotic treatment. Moreover, our work investigated if the beneficial effects brought about by the probiotics were age-related. This study provides new data for future development of probiotic-based products.

## Materials and Methods

### Probiotic Compound

A multi-strain probiotics compound was designed for use in this study. It was composed of equal proportion of three bacterial strains, namely *Lactobacillus casei* Zhang, *Lactobacillus plantarum* P-8, and *B. animalis* subsp. *lactis* V9, in a final concentration of 2 × 10^9^ CFU/g.

### Experimental Design and Canine Subjects

The use of experimental canine was permitted by canine owners, and all experimental procedures were approved by the Institute of Animal Science, Inner Mongolia Agricultural University of China. The experiment was performed between April and July 2016 (lasted 75 days) in a canine training base near Beijing, China. A total of 90 dogs (29 females, 25 males, and 36 of unknown gender) of different ages from this training base were enrolled as subjects (Table S1). The breeds of these dogs were mainly German Shepherd (57 dogs) and Belgium Shepherd (33 dogs). The 90 dogs were divided into three groups based on age and whether they attended training, i.e., the elderly (60–156 months old; stopped training due to age or injury; *n* = 30), young (<8 months old; not reached training age; *n* = 24), and training (9–24 months old; being trained for skills in searching objects, leaping over obstacles and so on; *n* = 36) groups, respectively. Each age group was further subdivided randomly into the probiotic treatment and control groups: treated elderly dogs (TO), control elderly dogs (CO), treated young dogs (TY), control young dogs (CY), treated training dogs (TT), and control training dogs (CT).

All the control subgroups were fed the base diet three times a day throughout the study period. All the treatment subgroups were fed the base diet supplemented with appropriate amount of compound probiotics (i.e., 10, 2, and 4 g/day for the old, young, and training groups, respectively) for 60 continuous days based on the weight and age of dogs (Table S1). The base diet was a commercially mixed dog food of the brands Royal Canin and Pedigree (purchased from Mars Inc., USA). The base diet consisted of 35% crude protein, 20% crude fat, 7% crude ash, and 2.5% crude fiber; all diets were nutritionally complete and contained more protein and fat than carbohydrate. None of the dogs had a history of antibiotic use or any other medication known to influence the intestinal microbiota for at least 3 months before and during the study.

Fresh fecal samples were collected at days 0 (pre-treatment period), 30, 60 (treatment period), and 15 days after ceasing probiotic treatment (post-treatment period) during morning dog walk. Each dog was walked individually to ensure correct sampling and minimize the chance of contamination between samples. All fecal samples were stored at −80°C until microbiota analysis. Meanwhile, blood samples were collected at the afore mentioned time points.

### Determination of Basal Physiological Parameters and Immune Markers

Some basal parameters of the dog subjects were monitored at days 0, 30, 60, and day 15 after stopping probiotic treatment, including the body weight, food consumption (the average daily feed intake (ADFI, the difference in animal weight before and after food ingestion), respiratory rate (the number of observed chest movements per minute), pulse rate (the number of beats per minute as detected via finger press on the femoral artery), and body temperature. All dogs were weighed individually at days 0, 30, 60, and day 15 after stopping probiotic treatment. The average daily weight gain (ADWG) between days 0 and 30, days 30 and 60, as well as day 60 and 15 days after ceasing probiotic treatment were calculated. A clinical score for diarrhea for each dog was recorded by trained assessors before the experiment started (day 0); the score took into consideration the stool consistency, defecation frequency, and volume of feces. The severity of diarrhea was graded as nil, mild, moderate, and severe, as represented by the scores 0, 3, 6, and 9, respectively (Table S1) (23). The diarrhea symptoms of each dog were monitored at 7 other time points (i.e., days 3, 7, 15, 30, 45, and 60 of treatment, and day 15 after stopping probiotic treatment).

All serum parameters were monitored at the baseline level (day 0), day 30, day 60, and day 15 after ceasing probiotic administration. White blood cell, lymphocyte, and neutrophil counts were analyzed at the Veterinary Medical Laboratory of Inner Mongolia Agricultural University using an automated blood-counter system. The serum concentrations of immunoglobulin G (IgG), tumoral necrosis factor-alpha (TNF-α), interleukin-6 (IL-6), and interferon-alpha (IFN-α) were measured using a sandwich enzyme-linked immunosorbent assay (ELISA) with the Quantikine Canine IgG, TNF-α, IL-6, and IFN-α kits (R&D Systems, Minneapolis, MN), respectively, according to the manufacturer's protocols.

The fecal secretory immunoglobulin A (SIgA) concentration was measured in the canine feces before (day 0), during (days 30 and 60), and after (day 75) probiotic administration using an ELISA validated for canine fecal IgA (24).

### Assessment of Intestinal Microbiota

Fecal samples were thawed before the extraction of genomic DNA with a QIAGEN DNA Stool Mini-Kit (QIAGEN, Hilden, Germany) according to the manufacturer's instructions (25). The quality of the extracted genomic DNA was verified by agarose gel electrophoresis and spectrophotometric analysis (optical density ratio at 260 nm/280 nm). All extracted DNA was stored at −20°C until polymerase chain reaction (PCR).

The PCR amplified the full-length bacterial 16S rRNA genes for SMRT barcode sequencing using the forward 27F (5′-AGAGTTTGATCMTGGCTCAG-3′) and reverse 1492R (5′-ACCTTGTTACGACTT-3′) primers (19). A distinct set of 16-base barcodes was added to the forward and reverse PCR primers to label each sample. Amplifications of DNA were performed as previously described (21). The PCR program was as follows: 95°C for 4 min; 28 cycles of 95°C for 30 s, 58°C for 30 s, and 72°C for 30 s with a final extension of 72°C for 5 min.

All the 16S rRNA gene amplicons were used for constructing DNA libraries with a PacBio SMRTbell™ template prep kit 1.0, as previously described (19). Sequencing was performed using P6-C4 chemistry on a PacBio RS II instrument (Pacific Biosciences), according to the manufacturer's instructions. The protocol RS_ReadsOfinsert.1 was used to process the raw data, which was available in the SMRT Portal version 2.7. Strict filtering criteria were applied: (i) a minimum of 5 full passes; (ii) a minimum predicted accuracy of 90%; and (iii) an insert read length of 1,400–1,800 bp (26).

Then, the filtered reads were sorted into different samples based on the barcode sequences. The bioinformatic analysis was performed on the high-quality sequences extracted using the Quantitative Insights into Microbial Ecology (QIIME) package (version 1.7). Briefly, the sequences were aligned by PyNAST (27) and clustered under 100% sequence identity by UCLUST (28). The unique sequence set was classified into operational taxonomic units (OTU) with a 98.65% threshold identity according to a previous study (29); and representative sequences were selected using UCLUST. Chimera Slayer was used to remove chimeric sequences from the representative OTU set (30). The taxonomy of each representative OTU sequence was assigned using the Ribosomal Database Project (RDP) classifier and the Greengenes database (version 13_8) with a minimum bootstrap threshold of 80% (31). Alpha and beta diversity were calculated based on a *de novo* taxonomic tree constructed by the representative chimera-checked OTU set using FastTree (32). To evaluate the sequence depth and biodiversity richness, the Shannon–Wiener, Simpson's diversity, Chao1, and rarefaction estimators were calculated. To assess the microbiota structure in different samples, principal coordinate analysis (PCoA) was performed based on the weighted UniFrac distance derived from the phylogenetic tree (33). Permutational multivariate analysis of variance (PERMANOVA) was performed based on the weighted Unifrac distance to assess which factors played significant role in shaping the variation of gut microbiota structure. Linear discriminant analysis (LDA) effect size (LEfSe) was used to identify differential abundant bacteria between any two groups with a statistically significant cut-off of LDA value of 2. The sequence data reported in this study have been deposited in the MG-RAST database (Accession No. 4737478.3 to 4737838.3).

### Effect of Probiotic Treatment on the Intestinal Microbiota Age Index of the Elderly Dogs

In order to assess the impact of probiotic application on the age of subjects' gut microbiota, here we built an age-predictive model based on the OTU-level microbiota profile of all 90 dogs at day 0 before probiotic treatment started. The age-predictive model was achieved by the machine learning algorithm Random Forests (regression against the chronological age of dogs) available in the R software (3.1.1) with the default parameters (34). The Random Forests algorithm ranked all the genera based on the 'feature importance' (i.e., age), determined the number of top-ranking age-discriminatory genera required for the prediction using the “rfcv” function over 100 iterations, and then computed the intestinal microbiota age index based on the relative abundance of the selected top-ranking age-discriminatory genera. A higher intestinal microbiota age index represented an 'older' microbiota. To evaluate the effect of probiotic treatment on the gut microbiota of the elderly dogs, their intestinal microbiota age index at days 30, 60 of probiotic treatment and day 15 after stopping probiotic intake was determined and compared with that of the elderly, young, and training groups at day 0 (baseline level).

### Statistical Analyses

All statistical analyses were performed with the R programming language (version 3.1.3). The pairwise Mann-Whitney test was used to detect any differential abundance between samples at the phylum, genus, and species levels. For all tests, a *P*-value < 0.05 was considered statistically significant for rejection of the null hypothesis. The Benjamini-Hochberg method was used for adjustment of false discovery rate (FDR). Moreover, the proportion of fecal bacteria at each phylogenetic level was compared independently (35). The graphs were generated by GraphPad Prism 6 and R package. Correlations between fecal bacteria and immune indexes were evaluated by using the Spearman rank correlation coefficient in R package; and Cytoscape 3.5.1 was used for network building.

## Results

### Probiotics Ameliorated Canine Health Status

**Figure 1:**
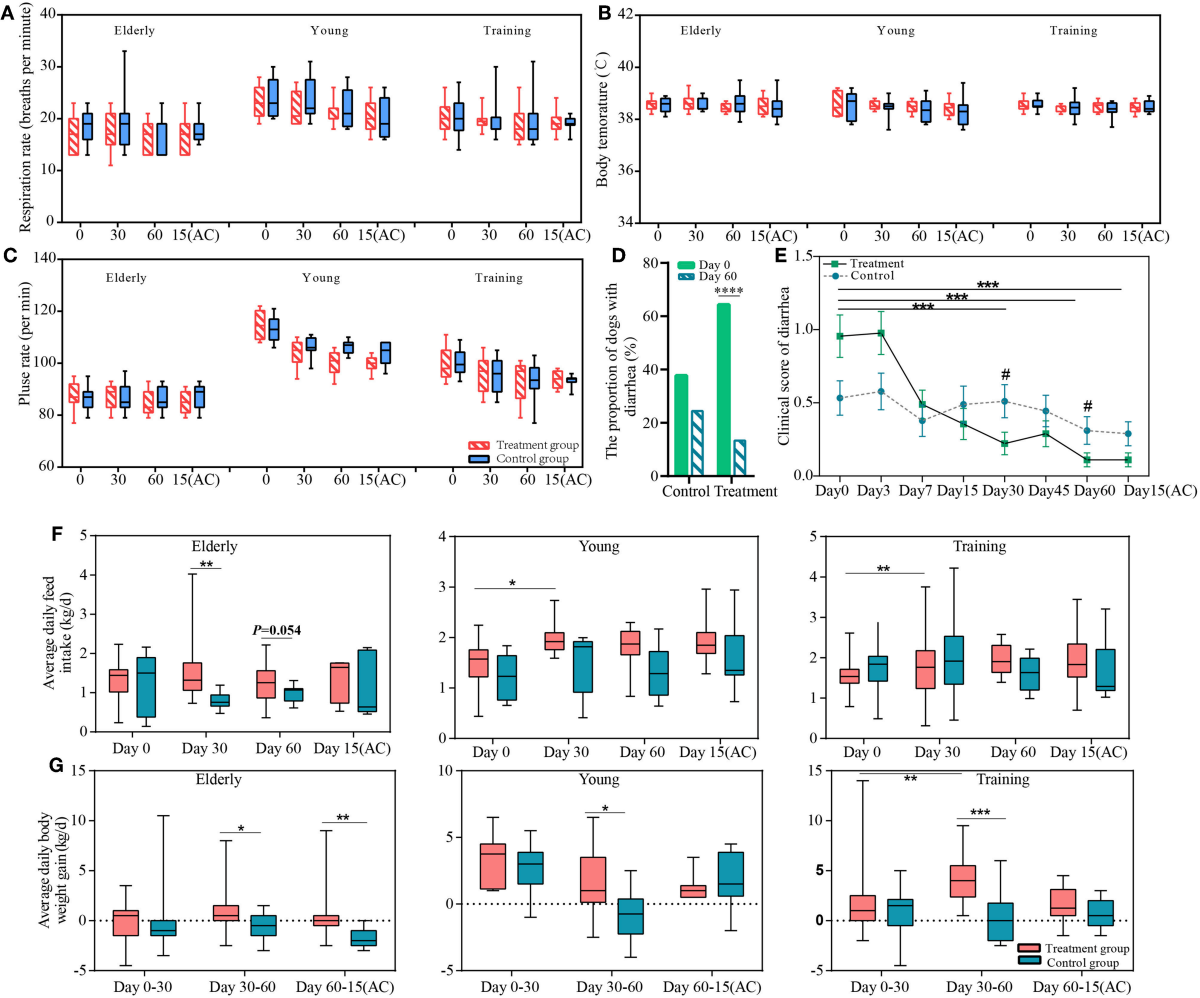
The effects of the probiotics compound administration on host health. Respiration rate **(A)**, body temperature **(B)**, pulse rate **(C)** of three sample groups (elderly, young, and training groups); **(D)** the proportion of dogs with diarrhea at day 0 and day 60 of probiotic administration; **(E)** Changes in severity of diarrhea of the control and probiotic treatment groups. Error bars represent SEM. ^“***”^ represents comparison between different time points of the treatment group, ^***^P < 0.001; “#” represents comparison between the probiotic treatment and control groups at the same time point #*P* < 0.05. The changes in average daily **(F)** feed intake and **(G)** weight gain of the elderly, young, and training dogs, with or without probiotic treatment. Parameters were monitored at days 0, 30, 60, and 15(AC) (15 days after ceasing probiotic treatment). ^*^P < 0.05, ^**^P < 0.01, and ^***^P < 0.001.

Firstly, we observed the effects of the probiotic treatment on host health. No significant difference was detected in the respiratory rate, pulse rate, body temperature, and white blood cell (including lymphocyte and neutrophil) counts between the probiotic and control groups (Figures 1A–C, Table S2), suggesting that the probiotic treatment did not cause any observable adverse physiological effects to the dogs and that it was safe for use in dogs. The diarrhea symptoms of all the dogs were assessed before and during the course of probiotic treatment (Figures 1D,E). the proportion of diarrhea in dogs who received probiotics at the end of the intervention was significantly lower than before the experiment (*P* < 0.0001). The probiotic use significantly lowered the clinical scores, i.e., diarrhea symptoms, of the probiotic-treated dogs at days 30 and 60 (compared with the non-treatment subjects); and the symptom relief effect lasted until 15 days after ceasing probiotic application. Moreover, the diarrhea clinical score was significantly lower for the probiotic receivers since the treatment started. These results together demonstrated the efficacy of the probiotic treatment in diarrhea alleviation.

Our results show positive effects of probiotic treatments on the canine feed intake and weight gain. The ADFI of the young and training dogs significantly increased after 30 days (vs. day 0) of probiotic administration (*P* < 0.05 and *P* < 0.01, respectively). Compared with the non-treated control group, the ADFI of the probiotic-receiving elderly dogs was significantly higher at day 30 (*P* < 0.05) (Figure 1F). The probiotic treatment also resulted in significant increase in ADWG in all three groups between days 30 and 60 (*P* < 0.05 for the elderly and the young groups, *P* < 0.001 for the training group). Moreover, it is interesting to note that the weight gain effect of the probiotic-receiving elderly dogs was still significantly higher between day 60 and 15 days after stopping probiotic treatment, suggesting such effect was long-lasting (Figure 1G).

### Probiotics Improved Canine Immune Responses

**Figure 2:**
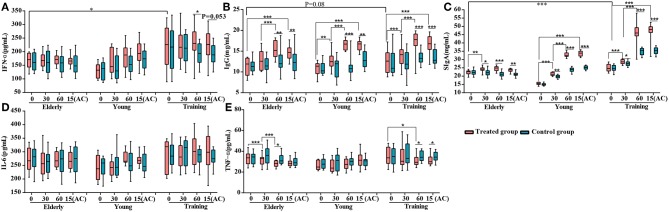
Effects of probiotic treatment on canine immune markers. The levels of **(A)** serum IFN-α, **(B)** serum IgG, **(C)** fecal secretory IgA (SIgA), **(D)** serum IL-6, and **(E)** serum TNF-α of the elderly, young, and training dogs, with or without probiotic treatment. Parameters were monitored at days 0, 30, 60, and 15(AC) (15 days after ceasing probiotic treatment). ^*^P < 0.05, ^**^P < 0.01, and ^***^P < 0.001.

To investigate the effects of the probiotic treatment on host immunity, the serum IFN-α, IgG, IL-6, and TFN-α, as well as fecal SIgA, were monitored. The level of serum IFN-α was only significantly enhanced in the training group at day 60 (*P* < 0.05) (Figure 2A). For all three groups, the probiotic treatment significantly increased the levels of serum IgG and fecal SIgA at multiple time points during the period of probiotic administration and 15 days after the discontinuation of probiotic administration, comparing with both the corresponding non-treatment control and the baseline level at day 0 (Figures 2B,C) However, the probiotic treatment did not result in significant change in the serum IL-6 level for all three groups (Figure 2D). The level of serum TFN-α was significantly lower in the receiving probiotic elderly and training groups at day 60 when compared with the control group, while no significant effect was observed for the young dogs regardless of probiotic treatment (Figure 2E). Finally, the level of fecal SIgA, serum IgG and IFN-α were apparently lower in the elderly group compared with the training group (Figures 2A–C).

### Difference in Different Age Canine Gut Microbiota

Although our data demonstrated that probiotics could exert ameliorative effects on canine health, we next investigated whether probiotics could influence the gut microbiota that may regulate host's health. A total of 2,434,051 full-length bacterial 16S rRNA sequence reads were produced, with an average of 6761.25 (range = 2,353–19,675, SD = 3249.00) sequence reads per sample. After quality filtering, the total number of unique and classifiable representative bacterial OTU sequences was 592,204 (average = 2975.14 OTUs per sample, range = 385–11,577, SD = 1466.81; Table S3). The Shannon index, Simpson diversity index, Chao1, and number of observed species of each sample were used to evaluate species richness and diversity (Table S3). These values suggested that most samples exhibited a high level of bacterial biodiversity.

**Figure 3:**
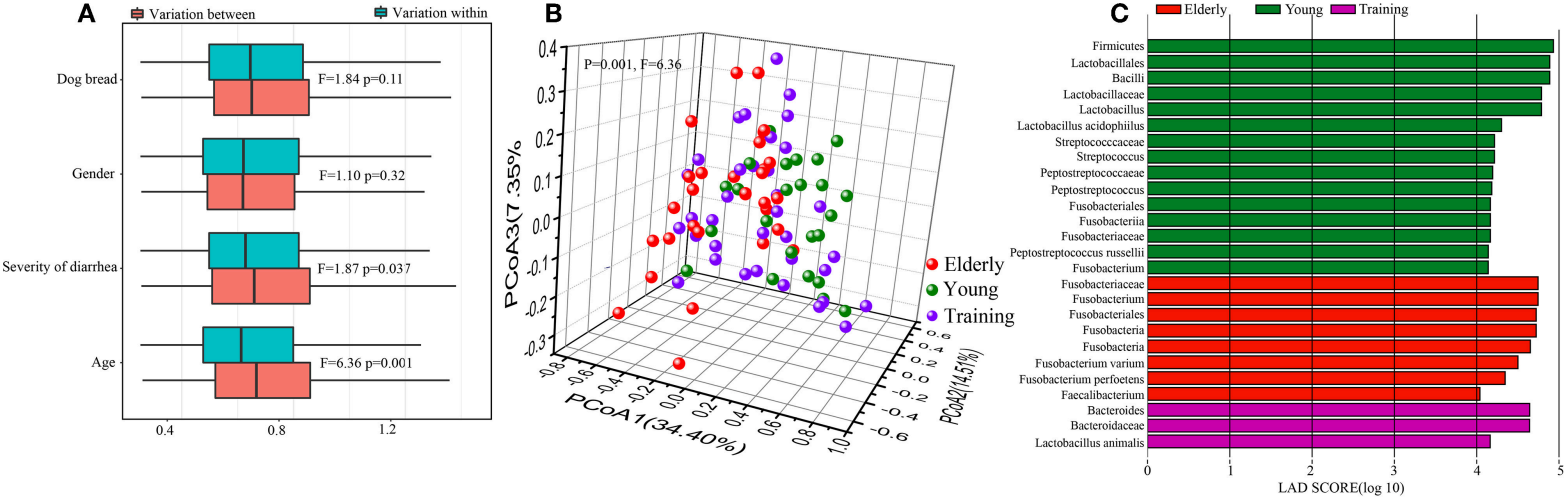
Difference in different age canine gut microbiota. **(A)** Contribution of canine breed, gender, severity of diarrhea, and animal age on the gut microbiota composition by permutational multivariate analysis of variance (PERMANOVA) based on the weighted Unifrac distance. Results for both within- and between-group variation are shown. **(B)** The principal coordinate analysis (PCoA) score plots of all canine subjects based on the weighted UniFrac distance. **(C)** Bar plots and cladograms showing differential abundant bacteria between the elderly dogs, young dogs, and training dogs, as identified by linear discriminant analysis (LDA) effect size (LEfSe). The LDA cut-off score was 4.

Firstly, to identify the influence of host factors, namely breed, gender, severity of diarrhea, and age, on the canine gut microbiota, PERMANOVA test (24) was performed based on the weighted UniFrac distance at the OTU level. Only data from the dog subjects of known sex were included in the calculation. The age of the dog subjects was found to be the most significant factor impacting the gut microbiota (*F* = 6.36, *P* = 0.001), followed by the severity of diarrhea (*F* = 1.87, *P* = 0.037). The dog breed (*F* = 1.84, *P* = 0.11) and gender (*F* = 1.10, *P* = 0.32) did not seem to contribute significantly in shaping the fecal microbiota (Figure 3A). On the basis of the weighted UniFrac distance from the 90 dogs at day 0, the overall structure of the gut microbiota showed significant difference among three groups (*F* = 6.36, *P* = 0.001) (Figure 3B), reflected a distinct structure of the gut microbiota in different age dogs. We then used LEfSe to identify differential abundant bacteria representing the three age groups (Figure 3C). Compared with the young and training groups, the elderly dogs had significantly more *Fusobacterium perfoetens* and *Fusobacterium varium*. Significantly more *Streptococcus, Peptostreptococcus russellii* and *Lactobacillus acidophilus* were detected in the young dogs. For all of training dogs, the genus *Bacteroides* and *Lactobacillus animalis* were remarkably enriched compared with the young dogs and elderly dogs.

### Effect of Probiotic Application on the Gut Microbiota Diversity and Structure

**Figure 4:**
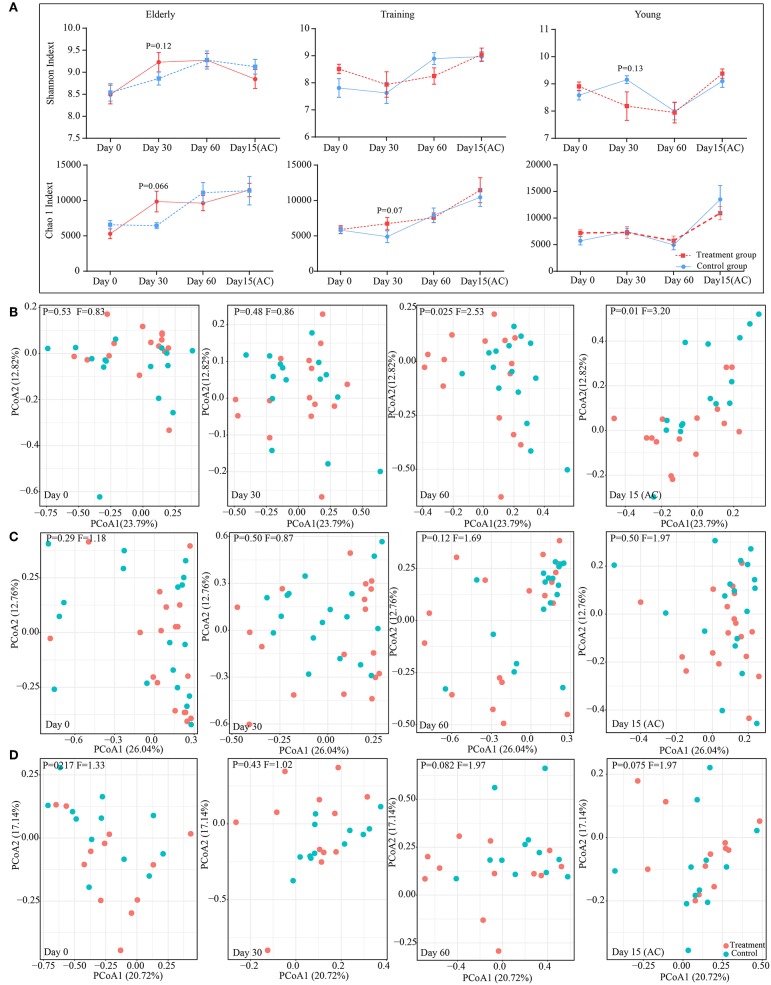
Effect of probiotic application on the gut microbiota diversity and structure. **(A)** The effect of probiotics on canine gut microbial α diversity. The principal coordinate analysis (PCoA) score plots of the elderly **(B)**, young **(C)**, and training **(D)** groups based on the weighted UniFrac distance days 0, 30, 60, and 15(AC) (15 days after ceasing probiotic treatment), respectively. *F*-value and *P*-value on the PCoA score plots represent the difference of two groups generated by PERMANOVA.

The changes in diversity and richness of the gut microbiota in dogs with different age during the administration of probiotics was indicated by the Shannon index and Chao 1 index, respectively (Figure 4A). There is no significant difference (*P* > 0.05) between control group and probiotics group for the Shannon index and the Chao 1 index during the experiment. However, there is a slightly higher in the levels of richness of intestinal microbiota (Chao 1 index) in receiving probiotics elderly (*P* = 0.066) and training dogs (*P* = 0.07) than dogs in control group at day 30 of probiotics use. The changes in canine gut microbial structure with the probiotics application were investigated based on the principal coordinates analysis (PCoA) of weighted UniFrac at the OTU level (Figures 4B–D). For the elderly dogs, probiotics treatment group and control group could be clearly distinguished at day 60 of probiotics use and at day 15 after ceasing probiotic administration, the significant difference in the gut microbial structure between two groups was observed at day 60 (*P* = 0.025, *F* = 2.53), and the difference persisted even after stopping probiotic administration for 15 days treatment (*P* = 0.01, *F* = 3.20) (Figure 4B). However, the training dogs displayed no significantly difference in structure of gut microbiota between probiotics group and control group during the whole experiment (Figure 4C). Additionally, the young dogs demonstrated a slight difference between probiotics group and control group at day 60 of administration of probiotics (*P* = 0.082, *F* = 1.97) and 15 day after ceased probiotic use (*P* = 0.075, *F* = 1.97) (Figure 4D). Gut microbial structure analysis showed that the changes in intestinal microbial structure of different age dogs was diverse during the probiotic application, especially the alteration in elderly dogs was more obvious. Therefore, we further explored whether the changes in composition of intestinal microbiota was age-related.

### Probiotic-Induced Several Changes in the Gut Microbial Composition

**Figure 5:**
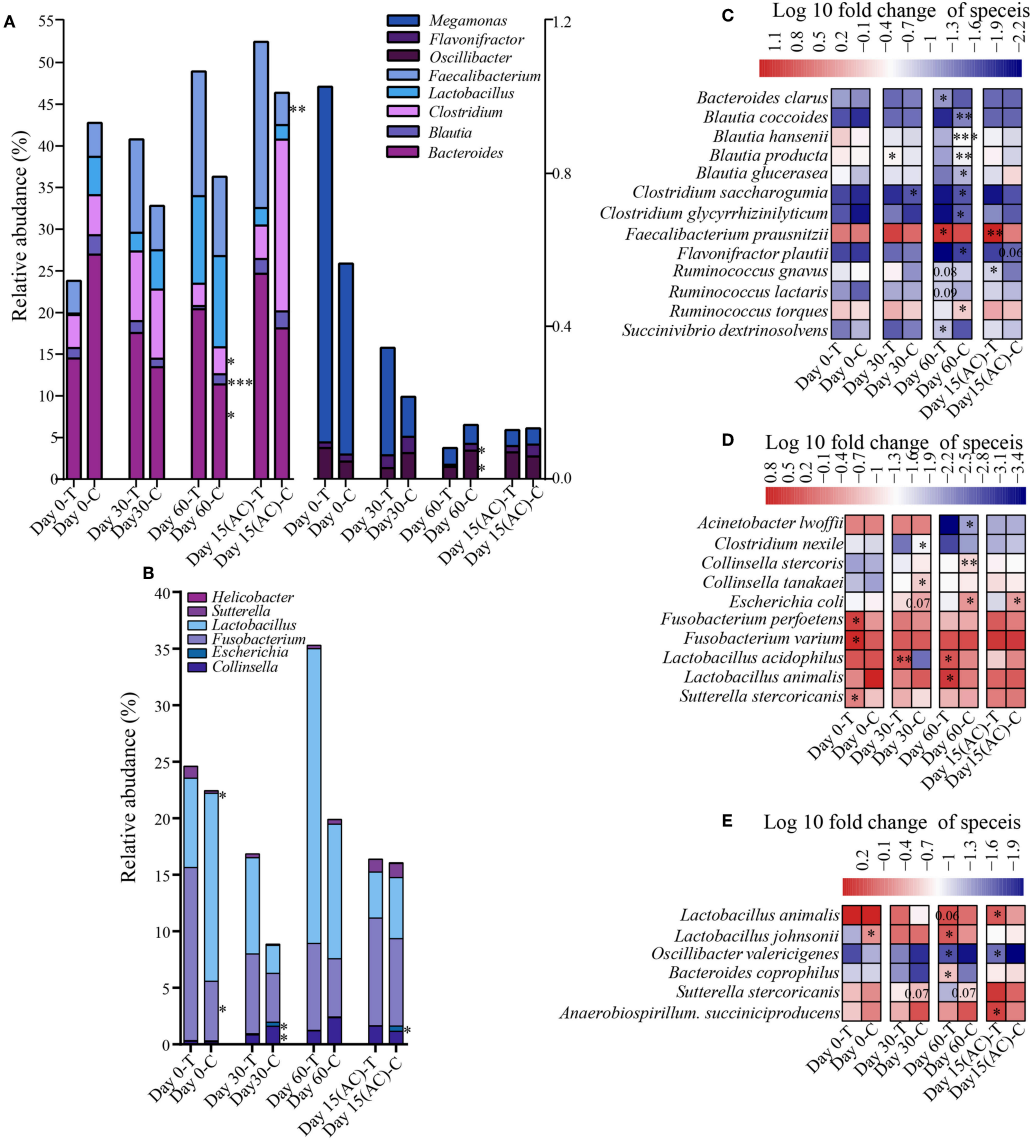
Effects of probiotic administration on the gut microbial composition. The average relative abundance of the differentially bacterial genus in elderly dogs **(A)** and **(B)** training dogs, respectively. Heatmaps of differential abundant bacterial species (significantly altered in relative abundance at least at one time point) modulated by probiotic treatment in the **(C)** elderly, **(D)** training, and **(E)** young group. ^*^P < 0.05, ^***^P < 0.01, and ^***^P < 0.001.

To assess how probiotic administration affected the canine gut microbial composition, the genus and species-level bacterial relative abundance of the control and treatment groups at each time point was compared (Figures 5A–E). At the genus level, there is no significant alteration in young dogs during the experiment. However, in the elderly dogs and training dogs, some interesting changes were observed. For instance, compared with control group, the *Bacteroides* and *Faecalibacterium* was increased in treatment group from day 0 to 15 day after ceased probiotic application, and the significantly higher abundance in *Bacteroides* (*P* < 0.05) in treatment group was detected at day 60, and the markedly more *Faecalibacterium* (*P* < 0.01) was detected at 15 day after ceased probiotic application. At day 60, there is significantly lower *Clostridium* (*P* < 0.05), *Flavonifractor* (*P* < 0.05), *Oscillibacter* (*P* < 0.05) and *Blautia* (*P* < 0.001) in the probiotics group than control group in elderly dogs. Moreover, for training dogs, the lower abundance in *Escherichia* (*P* < 0.05) at the end of the experiment was observed in probiotics group than control group. At the species level, there are still stronger response to probiotics in gut microbiota of elderly dogs. For example, significantly more *Faecalibacterium prausnitzii* was observed in the probiotic-treated group over time, and the increase persisted even after stopping probiotic administration for 15 days (*P* < 0.01). The proportion of *Bacteroides clarus* was also higher significantly in the probiotic treatment group than control group at day 60 (*P* < 0.05). Significantly less *Blautia coccoides* (*P* < 0.01), *Blautia hansenii* (*P* < 0.001), and *Blautia product* (*P* < 0.01) were detected at day 60 in the probiotic treatment group vs. the control group. The proportion of some *Clostridium* species diminished slightly, while the levels of *Succinivibrio dextrinosolvens* increased in the probiotic receivers compared with the control group, and there is slightly higher relative abundance in *Ruminococcus gnavus* (*P* = 0.08) and *Ruminococcus lactaris* (*P* = 0.08) but less *Ruminococcus torques* (*P* < 0.05) in treatment group than control group at day 60 of administrated probiotics (Figure 5C). For the probiotic-receiving training dogs, significantly more *Lactobacillus animalis* (*P* < 0.05) and *Lactobacillus acidophilus* (*P* < 0.05), while obviously less *Escherichia coli* (*P* < 0.05) and *Collinsella stercoris* (*P* < 0.01) was detected in the treatment group (vs. non-treatment control) at day 60. The relative abundance of several other species, including *Fusobacterium varium, Fusobacterium perfoetens*, and *Sutterella stercoricanisin*, decreased in the probiotic group at least at one time point (*P* < 0.05) (Figure 5D). More *Bacteroides coprophilus* (*P* < 0.01), *Lactobacillus animalis* (*P* = 0.06) and *Lactobacillus johnsonii* (*P* < 0.05) were detected in the fecal samples of the young probiotic-receiving dogs at day 60, while the decrease of *Sutterella stercoricanisin* was also observed in the probiotic-receiving young dogs (Figure 5E).

### Prolonged Probiotics Treatment Temporarily Reduced the Intestinal Microbiota Age Index of Elderly Dogs

**Figure 6:**
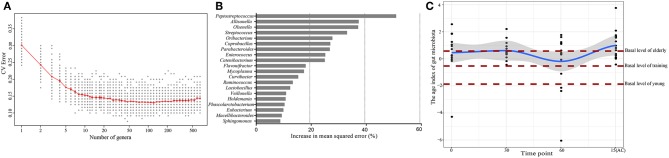
Effects of probiotic administration on the gut microbiota age index and composition. **(A)** Cross-validation error as a function of the number of input genus-level taxa used to regress against the age of dogs, in the order of variable importance. **(B)** The 20 top ranking age-discriminatory bacterial marker genera as identified by the Random Forests regression. **(C)** The age index of the gut microbiota of the probiotic-receiving elderly dogs at days 0, 30, 60, and 15(AC) (15 days after ceasing probiotic treatment) (linked by the blue line), as predicted by the Random Forests model. Each dot represents the gut microbiota age index of one dog subject. The brown lines mark the basal levels (at day 0) of the gut microbiota age index of the elderly, young, and training groups.

Since age was the most important factor that shaped the canine intestinal microbiota and the high responsiveness of the elderly dog gut microbiota upon probiotic treatment, we further characterized the probiotic-induced change in the gut microbiota aging state of the elderly group by the Random Forests regression model. The model regressed the relative abundance of all detected taxa against the chronological age of the canine subjects (Figure 6A). From the regression results, 20 age-discriminatory marker genera were identified (Figure 6B). Based on the composition of these 20 marker genera, the intestinal microbiota age index was calculated for each sample group at day 0. A low value of intestinal microbiota age index indicated a “young” intestinal microbiota structure. As expected, the intestinal microbiota age index of the elderly, training, and young groups at day 0 ranked in descending order. Interestingly, a 60-day probiotics treatment shifted the intestinal microbiota age index of the elderly groups toward that of the training dogs. However, such effect reversed 15 days after stopping probiotic treatment (Figure 6C).

### Effect of Probiotics on Bacteria Correlated With Feed Intake, Body Weight Gain and Immunity in Canine

**Figure 7:**
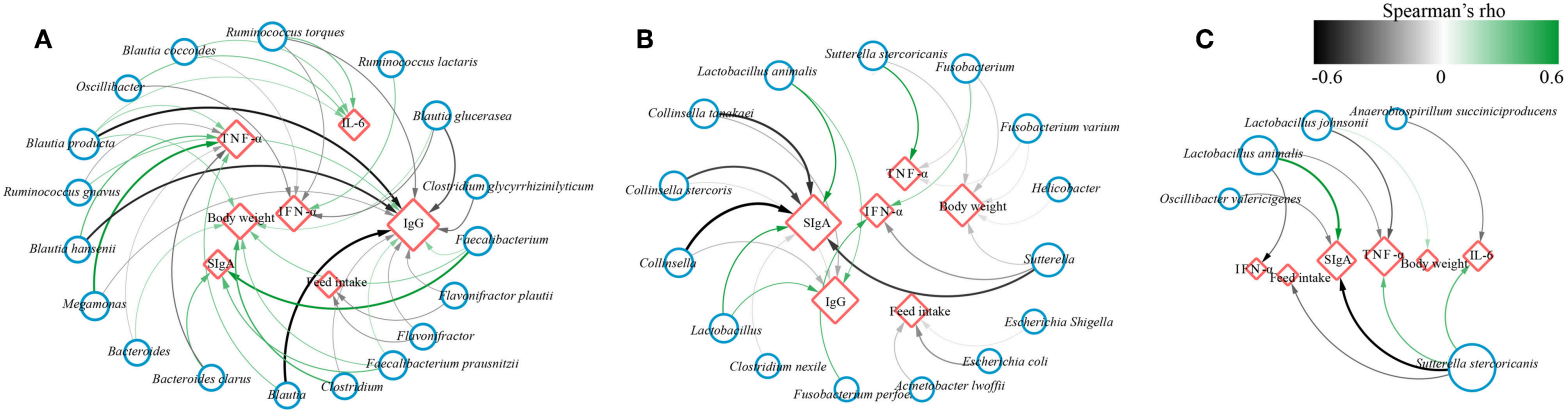
Network plot showing Spearman's correlation between the differentially bacteria and feed intake, body weight, immune indexes in **(A)** elderly dogs, **(B)** training dogs and **(C)** young dogs. Blue circles and red diamonds represent significantly correlated bacteria and physiological indices (feed intake, body weight, fecal secretory IgA, serum IgG, IFN-α, IL-6, and TNF-α), respectively. The size of the red diamonds corresponds to the number of significantly correlated bacteria. Significant correlations between the bacteria and immune factors, feed intake, body weight are connected by curve; the color of the curve lines represents the correlation strength as illustrated by the color scheme. The color scheme representing the Spearman's rho ranks between 0.6 and −0.6. Positive and negative Spearman's rho represent positive and negative correlation, respectively.

To investigate whether the effect of probiotics on gut microbiota was associated with improvement of canine health and enhancement of immunity, the correlation coefficients between the differentially abundant bacteria and the ADFI, body weight or immune factors using Spearman's rank correlation analyses in different age dogs (Figure 7). The results showed that some significant positive correlations were identified between the levels of fecal SIgA, serum IgG and body weight with the abundance of *Faecalibacterium, Faecalibacterium prausnitzii, Bacteroides*, and *Bacteroides clarus*, while the level of IgG was negatively correlated with the genus *Blautia* as well as its species *Blautia hansenii, Blautia producta*, the genus *Megamonas*, and *Ruminococcus torques*, and they were positively associated with TNF-α and IL-6. Additionally, the relative abundance of genus *Oscillibacter*, the species *Blautia coccoides, Ruminococcus torques* was negatively linked to IFN-α, and *Ruminococcus lactaris* was positively linked to IFN-α, and *Flavonifractor* and *Flavonifractor plautii* was negatively associated with feed intake in elderly dogs (Figure 7A). For training dogs, the level of fecal SIgA and serum IgG was positively correlated with the abundance of *Lactobacillus* and *Lactobacillus animalis*, whereas they were negatively correlated with the genus *Collinsella* and *Sutterella*. The *Helicobacter, Sutterella, Fusobacterium* and *Escherichia/Shigella* was negatively associated with feed intake or body weight, and the *Sutterella stercoricanis* was positively correlated with TNF-α (Figure 7B). For young dogs, the fecal SIgA was positively correlation with *Lactobacillus animalis*, while it was negatively correlation with *Oscillibacter valericigenes* and *Sutterella stercoricanis*. Inversely, *Sutterella stercoricanis* positively linked to TNF-α and feed intake, and *Lactobacillus animalis, Lactobacillus johnsonii* was negatively with TNF-α (Figure 7C). These results indicated that intestinal microbiota might be linked to canine ADFI, ADWG and immunity and that probiotics improved canine health and immunity, possibly by regulating certain specific bacteria in the gut.

## Discussion

The gut microbiota is closely linked to the host health; and the consumption of probiotics confer numerous beneficial effects to the host. However, only limited studies have focused on how probiotic consumption improves the health of different age host. Thus, the current work investigated the beneficial effects of feeding a probiotic compound to different age dogs. We further investigated whether such effects were age-related.

Firstly, we verified the safety of the probiotics for canine through monitoring some physiological parameters such as body temperature and white blood cell and found the probiotics compound could effectively alleviate the clinical symptoms of diarrhea in dogs. Interestingly, we observed that only the ADFI of the elderly dogs (but not the young and training groups) significantly increased after 30 days of probiotics administration, although the overall ADFI of the elderly dogs was still lower than that of the other groups. The ADWG of all groups significantly increased between day 30 and day 60; however, only in the elderly group, the effect lasted until 15 days after ceasing probiotic treatment. These observations might indicate probiotics can improve the canine health such as the ADFI and ADWG to a certain degree, especially for the elderly dogs.

The gut serves as an immune barrier that protects the body from toxins, bacteria, and other potential damages. To investigate the probiotic-induced immunity modulation in the dog subjects, our work analyzed changes of several immune markers, namely fecal SIgA, serum IgG, IFN-α, TFN-α, and IL-6. SIgA is the dominant antibody type found in mucosal secretions, and it has long been considered as the first-line immune defense that resists invading pathogens at the mucosa (36). The antibody preferentially targets bacteria that colonize the gut mucosal-proximal surface. It can prevent bacteria and viruses from adhering to the surface of epithelial cells and neutralize toxins/foreign antigens (37). The serum IgG can enter the intestinal lumen serum and bind to Gram-negative bacteria to lower the risks of bacterial translocation, intestinal damage, and systemic infection (38). Here, we observed significantly higher concentrations of fecal SIgA and serum IgG in the probiotic-treated dogs compared with the control. Also, the level of IFN-α increased mildly in the probiotic treatment groups, and it was significantly higher in the receiving probiotic training group at day 60. Interferon-α combats viruses by mechanisms independent from B and T cell responses; and it controls systemic viral replication via the JAK/STAT signaling pathway (39). Moreover, although the levels of fecal SIgA, serum IgG and IFN-α in elderly dogs were lower than that of the training dogs at day 0, their concentrations increased after probiotic treatment, indicating that probiotic ingestion could boost immunity of the elderly dogs. In contrast, the serum TNF-α level of the elderly and training dogs significantly reduced after probiotic administration, especially for probiotics elderly group. TNF-α is a classical pro-inflammatory cytokine, which is enhanced by lipopolysaccharide (LPS), a main component of the cell wall of Gram-negative bacteria. We found no significant difference in the serum IL-6 level between the probiotic and the control groups. Similar observations were reported by Liu et al. that probiotic treatment did not influence the level of IL-6 in postoperative colorectal cancer patients (40). These results suggest that the probiotic treatment could strengthen the gut immune barrier function by increasing some immune factors and reducing the levels of pro-inflammatory cytokines.

The gut microbiome has been demonstrated to play a crucial role in the maintenance of many aspects of health, especially immunity. Therefore, we further investigated how probiotics could influence the gut microbiota, whether these changes in gut microbiota is related with host's health and immunity. Based on PERMANOVA analysis, we revealed that dog age contributed most significantly for gut microbial composition, hence, we further analyzed whether exist the difference in the effect of probiotics on different age dogs.

Our results revealed more apparent changes in the gut microbial diversity and structure of the elderly dogs compared with the training dogs and young dogs, suggesting the probiotics compound could change the gut environment and microbiota of the elderly dogs. Researchers have observed that gut microbial structure and composition change as the host ages, although the precise mechanisms underlying the association of aging and gut microbiome are still unclear (41).

Furthermore, we explored the effect of probiotics on the taxonomy of different age dogs and observed the most alteration in bacterial community of elderly dogs. The results showed the probiotic administration remarkably increased the relative abundance of *Bacteroides* and *Faecalibacterium prausnitzii* and decreased some *Blautia* species including *Blautia hansenii* and *Blautia producta*, the genus *Oscillibacter, Megamonas, Flavonifractor*, as well as *Clostridium saccharogumia, Ruminococcus torques* in the elderly canine gut microbiota. *Bacteroides* is normal indigenous intestinal bacteria, and more *Bacteroides* was present in the training dogs (vs. the young and elderly dogs), which contributes to fermentative metabolism and degradation of oligosaccharides derived from plant-food (42). *Faecalibacterium prausnitzii* is a desirable bacterium that generates and supplies butyrate to the colonic epithelium; and it alleviates gut problems like inflammatory bowel disease and diarrhea (43, 44). *Blautia* produces acetic acid which has adverse effects on the intestinal tract, for instance, it may be a primary contributor to non-alcoholic fatty liver disease (45). *Ruminococcus torques* is a mucin degradation bacterium and *Oscillibacter* has been associated with high fat diet, which were relatively enriched in patients with age-related macular degeneration (46). Additionally, the abundance of these increased bacteria after administration of probiotics correlated strongly and positively with SIgA, IgG, and body weight, such as *Faecalibacterium prausnitzii*, and negatively associated with TNF-α, such as *Bacteroides*. Inversely, some decreased bacteria, such as *Blautia producta* and *Ruminococcus torques*, correlated negatively with the fecal SIgA and serum IgG. These findings suggest that the probiotic treatment could heighten some immune factors and decline the level of inflammatory through increasing the intestinal beneficial microbiota while suppressing potential pathogens. Beneficial microbes may enhance the gut function via producing certain metabolites. For example, short-chain fatty acids originated from gut microbes can promote B-cell differentiation into plasma cells, increase B-cell metabolism, boost B-cell glycolytic activity, and enhance host antibody production (47).

Similarly, the probiotic administration also modulated the gut microbiota composition of the training and young dogs. The relative abundance of *Lactobacillus animalis* and *Lactobacillus acidophilus* increased in training canine. In contrast, the relative abundance of some *Collinsella* species decreased in training dogs; the genus *Collinsella* is known to correlate with the host lipid metabolism (48). The gut microbiome of Mongolians was found to correlate with the abundance of *Collinsella*, and the typical Mongolian diet is rich in protein (49), suggesting that the growth of this genus is likely enhanced by a high protein content. Consistently with these results, the canine diet was high in both protein and fat, and our data implicated that the applied probiotic strains might help control the growth of *Collinsella*. Less *Sutterella stecoricanis* and *Escherichia coli* was detected in the probiotic-treated training group. It has been confirmed that some members of the *Sutterella* genus could lower the intestinal SIgA level by degrading both SIgA and SIgA-stabilizing peptide (50). Meanwhile, we also detected the *Collinsella, Sutterella* as well as *Escherichia coli* were negatively associated with canine feed intake, body weight, as well as SIgA and IgG, and positively associated with TNF-α. For young dogs, the relative abundance of *Bacteroides coprophilus, Lactobacillus animalis* and *Lactobacillus johnsonii* increased and *Sutterella stercoricanis* also significantly reduced after probiotics use. The correlation between altered bacteria and the feed intake, body weight and some immune factors, also showed the *Sutterella stercoricanis* might contribute to reduce the fecal SIgA, and *Lactobacillus* might improve body weight and immunity in young dogs. However, we also observed the alteration in gut microbiota in different age dogs was distinct during the administration of this probiotics compound, suggesting that the effects of the probiotics product on hosts were age-related. Moreover, the gut microbiota in the elderly dogs showed the strongest response to the probiotics compared with the young dogs and training dogs. Hence, we further explored the changes of intestinal flora in elderly dogs.

The Random Forests algorithm was then applied to predict a panel of age-discriminatory genera and calculate the gut microbiota age index that reflects the maturity of the gut microbiota. The model predicted 20 age-discriminatory genera that belonged to the phyla Firmicutes, Actinobacteria, and Bacteroidetes. *Peptostreptococcus* was identified to be the top age-discriminatory genus; it plays important role in protein metabolism (51). Considering the important function of this genus in protein digestion, the high protein content in canine diet, and its differential distribution between the fecal microbiota composition of old and young dogs, we speculate that the aging of intestinal microbiota could be related with the food digestion ability. Moreover, we found an apparent decrease in the gut microbiota aging index after 60 days of probiotic application, approaching to the basal value of the training dogs. Such results suggest the shifting of the elderly gut microbiota to resemble that of the younger dogs. *Lactobacillus* was another age-discriminatory genus predicted by the Random Forests model; some *Lactobacillus* increase after oral administration of probiotics might have contributed to the decrease in the intestinal microbiota age index. A previous study has shown that feeding probiotics to broiler chicken modulated the gut microbiota and greatly enriched the lactobacilli diversity (16). Meanwhile, it was consistent with the results in significant increase in ADFI and ADWG and significant decrease in TNF-α level in the elderly dogs. These observations may suggest that the young-like gut bacterial community could improve the food digestion ability of the elderly dogs by modulating their appetite and nutrient assimilation. Age-related changes in gut microbiota are associated with increased inflammation and weakened immunity, and these changes may shorten animal life span in the long run (52). On the other hand, a healthy gut microbiota may salvage or slow down the aging process by preserving the innate immune homeostasis and eventually promoting longevity (53).

In conclusion, the probiotic administration was effective in improving canine feeding intake, and weight gain, immunity and the gut microbiota. Particularly, the oral probiotic supplementation could be a promising way to strengthen the gut mucosal immune barrier through gut microbiota modulation. Meanwhile, the elderly dogs showed the strongest response to the probiotics, and the probiotic application shifted the gut microbiota of older dogs to a young-like composition. Thus, we believe that the probiotic improvement of health and enhancement of immunity were age-related. Our work has provided interesting insights into the application of probiotics and future development of probiotic-based strategies to improve animal health.

## Author Contributions

HZ and ZS designed the study. WH, HX, WL, HM, and YW collected the samples and performed the experiments. HX and QH analyzed the data. HX wrote the manuscript. L-YK revised the manuscript.

### Conflict of Interest Statement

The authors declare that the research was conducted in the absence of any commercial or financial relationships that could be construed as a potential conflict of interest.
