# Supplementary material for: Oral Administration of Compound Probiotics Improved Canine Feed Intake, Weight Gain, Immunity and Intestinal Microbiota
Source: Front Immunol. 2019 Apr 2;10:666. doi: 10.3389/fimmu.2019.00666 (PMC6454072; doi:10.3389/fimmu.2019.00666)
Supplement: Supplementary file 1 [file Table_1.DOCX]

Table S1 Characteristics of the dogs enrolled in this study

| Dog ID | Group | Breed | Age (months) | Gender | Weight (kg) | Daily dose of  probiotics (g) | Severity of diarrhea^*^ |
| --- | --- | --- | --- | --- | --- | --- | --- |
| 1 | TO | German shepherd dog | 84 | Female | 41 | 10 | Moderate |
| 2 | TO | German shepherd dog | 72 | Male | 42 | 10 | Nil |
| 3 | TO | Wolf black | 72 | Female | 40.5 | 10 | Mild |
| 4 | TO | German shepherd dog | 96 | Male | 44.5 | 10 | Mild |
| 5 | TO | German shepherd dog | 84 | Male | 42 | 10 | Nil |
| 6 | TO | German shepherd dog | 84 | Female | 35 | 10 | Mild |
| 7 | TO | German shepherd dog | 120 | Female | 33.5 | 10 | Nil |
| 8 | TO | German shepherd dog | 84 | Female | 36.5 | 10 | Mild |
| 9 | TO | German shepherd dog | 96 | Female | 29.5 | 10 | Nil |
| 10 | TO | German shepherd dog | 72 | Male | 27 | 10 | Nil |
| 11 | TO | German shepherd dog | 96 | Female | 25.5 | 10 | Nil |
| 12 | TO | German shepherd dog | 84 | Female | 35 | 10 | Nil |
| 13 | TO | German shepherd dog | 108 | Male | 32 | 10 | Moderate |
| 14 | TO | German shepherd dog | 60 | Female | 43 | 10 | Nil |
| 15 | TO | German shepherd dog | 72 | Female | 42 | 10 | Nil |
| 16 | CO | German shepherd dog | 72 | Female | 36 | 10 | Mild |
| 17 | CO | German shepherd dog | 84 | Male | 43.5 | 10 | Nil |
| 18 | CO | Belgium shepherd dog | 96 | Female | 35.5 | 10 | Nil |
| 19 | CO | German shepherd dog | 96 | Male | 44 | 10 | Nil |
| 20 | CO | German shepherd dog | 60 | Male | 39.5 | 10 | Mild |
| 21 | CO | Labrador | 156 | Male | 26.5 | 10 | Moderate |
| 22 | CO | German shepherd dog | 96 | Female | 33 | 10 | Mild |
| 23 | CO | German shepherd dog | 72 | Female | 35.5 | 10 | Moderate |
| 24 | CO | German shepherd dog | 72 | Female | 41 | 10 | Nil |
| 25 | CO | German shepherd dog | 60 | Male | 42 | 10 | Nil |
| 26 | CO | German shepherd dog | 84 | Female | 31 | 10 | Nil |
| 27 | CO | Belgium shepherd dog | 60 | Female | 32 | 10 | Moderate |
| 28 | CO | German shepherd dog | 60 | Male | 36.5 | 10 | Nil |
| 29 | CO | German shepherd dog | 72 | Female | 25.5 | 10 | Nil |
| 30 | CO | German shepherd dog | 108 | Female | 38.5 | 10 | Nil |
| 31 | TY | Belgium shepherd dog | 6 | Female | 12.5 | 2 | Mild |
| 32 | TY | Belgium shepherd dog | 6 | Male | 16 | 2 | Mild |
| 33 | TY | Belgium shepherd dog | 6 | Female | 18.5 | 2 | Nil |
| 34 | TY | Belgium shepherd dog | 6 | Female | 14 | 2 | Nil |
| 35 | TY | German shepherd dog | 6 | Male | 19 | 2 | Moderate |
| 36 | TY | Hollandse Herdershond | 7 | Male | 18 | 2 | Moderate |
| 37 | TY | German shepherd dog | 5 | Male | 22.5 | 2 | Severe |
| 38 | TY | German shepherd dog | 5 | Female | 16.5 | 2 | Severe |
| 39 | TY | German shepherd dog | 4 | Male | 17.5 | 2 | Nil |
| 40 | TY | Hollandse Herdershond | 7 | Female | 14 | 2 | Nil |
| 41 | TY | German shepherd dog | 5 | Female | 20 | 2 | Mild |
| 42 | TY | German shepherd dog | 4 | Male | 12.5 | 2 | Mild |
| 43 | CY | Belgium shepherd dog | 6 | Female | 16 | 2 | Mild |
| 44 | CY | Belgium shepherd dog | 6 | Male | 15.5 | 2 | Nil |
| 45 | CY | Belgium shepherd dog | 6 | Female | 11.5 | 2 | Nil |
| 46 | CY | Belgium shepherd dog | 6 | Female | 14.5 | 2 | Nil |
| 47 | CY | German shepherd dog | 6 | Male | 20.5 | 2 | Nil |
| 48 | CY | Hollandse Herdershond | 7 | Male | 18 | 2 | Nil |
| 49 | CY | German shepherd dog | 6 | Male | 9 | 2 | Moderate |
| 50 | CY | German shepherd dog | 4 | Male | 18 | 2 | Mild |
| 51 | CY | German shepherd dog | 5 | Female | 18.5 | 2 | Mild |
| 52 | CY | German shepherd dog | 6 | Male | 18.5 | 2 | Mild |
| 53 | CY | German shepherd dog | 7 | Male | 26.5 | 2 | Moderate |
| 54 | CY | Belgium shepherd dog | 6 | Male | 12.5 | 2 | Mild |
| 55 | TT | Belgium shepherd dog | 17 | not recorded | 26 | 4 | Severe |
| 56 | TT | Belgium shepherd dog | 17 | not recorded | 23 | 4 | Mild |
| 57 | TT | German shepherd dog | 24 | not recorded | 17.5 | 4 | Nil |
| 58 | TT | German shepherd dog | 19 | not recorded | 39 | 4 | Nil |
| 59 | TT | German shepherd dog | 19 | not recorded | 28 | 4 | Moderate |
| 60 | TT | Belgium shepherd dog | 12 | not recorded | 24.5 | 4 | Mild |
| 61 | TT | German shepherd dog | 24 | not recorded | 32 | 4 | Mild |
| 62 | TT | Belgium shepherd dog | 24 | not recorded | 19 | 4 | Mild |
| 63 | TT | Belgium shepherd dog | 17 | not recorded | 21.5 | 4 | Mild |
| 64 | TT | Belgium shepherd dog | 17 | not recorded | 25.5 | 4 | Mild |
| 65 | TT | Belgium shepherd dog | 14 | not recorded | 17 | 4 | Mild |
| 66 | TT | German shepherd dog | 13 | not recorded | 33 | 4 | Mild |
| 67 | TT | German shepherd dog | 17 | not recorded | 35.5 | 4 | Mild |
| 68 | TT | Belgium shepherd dog | 12 | not recorded | 18 | 4 | Mild |
| 69 | TT | German shepherd dog | 12 | not recorded | 29.5 | 4 | Nil |
| 70 | TT | German shepherd dog | 17 | not recorded | 24 | 4 | Severe |
| 71 | TT | Belgium shepherd dog | 12 | not recorded | 17 | 4 | Nil |
| 72 | TT | German shepherd dog | 12 | not recorded | 16.5 | 4 | Severe |
| 73 | CT | Belgium shepherd dog | 17 | not recorded | 24.5 | 4 | Nil |
| 74 | CT | Belgium shepherd dog | 12 | not recorded | 16 | 4 | Nil |
| 75 | CT | German shepherd dog | 24 | not recorded | 38.5 | 4 | Nil |
| 76 | CT | German shepherd dog | 24 | not recorded | 35 | 4 | Nil |
| 77 | CT | Belgium shepherd dog | 12 | not recorded | 26.5 | 4 | Mild |
| 78 | CT | German shepherd dog | 24 | not recorded | 34 | 4 | Mild |
| 79 | CT | Belgium shepherd dog | 17 | not recorded | 22 | 4 | Mild |
| 80 | CT | Belgium shepherd dog | 12 | not recorded | 17 | 4 | Severe |
| 81 | CT | Belgium shepherd dog | 17 | not recorded | 22 | 4 | Nil |
| 82 | CT | Belgium shepherd dog | 9 | not recorded | 19.5 | 4 | Nil |
| 83 | CT | German shepherd dog | 12 | not recorded | 33.5 | 4 | Nil |
| 84 | CT | German shepherd dog | 12 | not recorded | 22.5 | 4 | Nil |
| 85 | CT | German shepherd dog | 24 | not recorded | 33.5 | 4 | Nil |
| 86 | CT | Belgium shepherd dog | 12 | not recorded | 17.5 | 4 | Nil |
| 87 | CT | German shepherd dog | 12 | not recorded | 33 | 4 | Nil |
| 88 | CT | German shepherd dog | 15 | not recorded | 23.5 | 4 | Nil |
| 89 | CT | German shepherd dog | 17 | not recorded | 29 | 4 | Nil |
| 90 | CT | German shepherd dog | 12 | not recorded | 23 | 4 | Nil |

Note: TO, Probiotics treated elderly dogs; CO, Control elderly dogs; TY, Probiotics treated young dogs; CY, Control young dogs; TT: Probiotics treated training dogs; CT, Control training dogs. *The severity of diarrhea was classified into 'nil', 'mild', 'moderate', and 'severe' based on stool consistency, defecation frequency, and volume of feces.

Table S2 White blood cell counts of different groups at different time points

| Groups | | | Leukocytes  (10^9^ cells/L) | Lymphocytes  (10^9^ cells/L) | Neutrophils  (10^9^ cells/L) |
| --- | --- | --- | --- | --- | --- |
| Elderly group | Day 0 | Probiotics treated | 16.2±5.3 | 5.4±1.6 | 11.3±2.8 |
|  |  | Control | 15.4±6.5 | 4.6±1.3 | 10.6±4.8 |
|  | Day 30 | Probiotics treated | 15.3±6.6 | 6.0±2.8 | 10.2±4.0 |
|  |  | Control | 13.4±3.4 | 4.5±2.0 | 8.6±3.6 |
|  | Day 45 | Probiotics treated | 15.2±11.5 | 6.4±2.5 | 9.2±2.5 |
|  |  | Control | 16.7±3.7 | 6.4±2.1 | 9.7±3.1 |
|  | Day 60 | Probiotics treated | 15.8±3.3 | 6.6±1.9 | 9.7±2.8 |
|  |  | Control | 14.9±4.2 | 5.1±2.4 | 8.9±3.3 |
| Young group | Day 0 | Probiotics treated | 21.9±3.8 | 7.2±2.2 | 12.2±3.4 |
|  |  | Control | 23.5±6.0 | 7.9±2.2 | 13.6±5.4 |
|  | Day 30 | Probiotics treated | 21.9±3.6 | 6.8±2.3 | 12.4±3.1 |
|  |  | Control | 22.0±6.5 | 6.9±2.8 | 13.2±4.5 |
|  | Day 45 | Probiotics treated | 23.0±5.2 | 9.0±4.4 | 11.3±3.4 |
|  |  | Control | 23.9±8.3 | 8.2±4.4 | 13.1±6.5 |
|  | Day 60 | Probiotics treated | 24.1±6.1 | 8.0±3.1 | 12.5±1.9 |
|  |  | Control | 26.7±11.0 | 8.2±5.0 | 15.6±6.8 |
| Training group | Day 0 | Probiotics treated | 20.1±7.4 | 6.9±3.2 | 11.8±3.3 |
|  |  | Control | 19.0±4.4 | 6.4±2.5 | 11.9±3.3 |
|  | Day 30 | Probiotics treated | 25.5±12.3 | 9.4±5.6 | 14.7±7.6 |
|  |  | Control | 22.4±7.6 | 7.0±4.3 | 13.3±5.7 |
|  | Day 45 | Probiotics treated | 20.3±4.8 | 8.4±3.0 | 11.8±3.4 |
|  |  | Control | 19.2±3.3 | 6.8±3.0 | 11.7±2.9 |
|  | Day 60 | Probiotics treated | 20.4±6.2 | 8.9±4.3 | 10.8±3.7 |
|  |  | Control | 19.6±4.4 | 7.8±3.0 | 10.7±3.0 |

Table S3 Sequence abundance and microbial diversity

| Sample | Day 0 | | | | Day 30 | | | | Day 60 | | | | Day 15 (AC*) | | | |
| --- | --- | --- | --- | --- | --- | --- | --- | --- | --- | --- | --- | --- | --- | --- | --- | --- |
|  | No. of reads | No. of OTU | Shannon index | Observed species | No. of reads | No. of OTU | Shannon index | Observed species | No. of reads | No.of OTU | Shannon index | Observed species | No. of reads | No.of OTU | Shannon index | Observed species |
| 1 | 6223 | 1996 | 7.4072 | 788.72 | 6320 | 2220 | 7.93497 | 874.5 | 6645 | 3396 | 9.33349 | 1229.36 | 5188 | 2805 | 8.85065 | 1204.18 |
| 2 | 7581 | 3087 | 8.72173 | 1029.52 | 8165 | 3235 | 8.69628 | 1018.3 | 7850 | 4484 | 9.71909 | 1369.4 | 6706 | 2395 | 8.18341 | 891.78 |
| 3 | 4703 | 2459 | 8.86879 | 1166.24 | 7092 | 3172 | 8.87506 | 1108.32 | 4636 | 1711 | 8.00281 | 870.04 | 8029 | 3747 | 9.18527 | 1149.8 |
| 4 | 13075 | 5213 | 8.91107 | 1081.52 | 17968 | 6846 | 8.97848 | 1091.4 | 14972 | 3855 | 8.38537 | 849.82 | 6241 | 3310 | 9.46397 | 1241.66 |
| 5 | 4584 | 1819 | 8.4346 | 929.1 | 11808 | 5500 | 8.9907 | 1184.24 | 7205 | 1742 | 7.22991 | 657.82 | 5666 | 3105 | 8.67475 | 1202.08 |
| 6 | 3663 | 1886 | 8.9469 | 1132.72 | 6404 | 3488 | 9.35341 | 1250.8 | 6006 | 3709 | 9.34535 | 1365.76 | 3691 | 2307 | 9.54525 | 1337.62 |
| 7 | 4712 | 2536 | 9.29335 | 1227.92 | 5800 | 2254 | 8.04529 | 956.66 | 5761 | 2736 | 9.41972 | 1173.22 | 3200 | 2479 | 10.2142 | 1606.74 |
| 8 | 6178 | 2632 | 8.34608 | 1002.88 | 2605 | 1453 | 9.49031 | 1177.86 | 5156 | 3590 | 9.90632 | 1520.22 | 3031 | 1085 | 8.15068 | 794.88 |
| 9 | 7789 | 2352 | 7.29393 | 766.1 | 6533 | 2380 | 7.94862 | 863.3 | 16710 | 9925 | 10.0784 | 1519.74 | 15321 | 4456 | 8.85091 | 940.04 |
| 10 | 6225 | 3329 | 9.68314 | 1274.26 | 8763 | 4066 | 9.03306 | 1153.52 | 6239 | 3769 | 9.67704 | 1369.4 | 6560 | 3461 | 9.3566 | 1228.24 |
| 11 | 6339 | 1955 | 7.29689 | 741.5 | 4296 | 2243 | 9.43683 | 1202.04 | 3936 | 2211 | 9.30851 | 1233.94 | 5947 | 4002 | 9.54585 | 1455.92 |
| 12 | 6868 | 2701 | 8.82918 | 1002.86 | 8876 | 3772 | 9.13441 | 1106.36 | 6369 | 3804 | 9.69835 | 1374.66 | 4247 | 3415 | 10.2601 | 1674.8 |
| 13 | 5007 | 2830 | 9.2804 | 1272.34 | 3680 | 2149 | 9.75422 | 1294.74 | 4517 | 3109 | 9.79188 | 1472.5 | 4106 | 2327 | 9.29886 | 1242.32 |
| 14 | 6285 | 3302 | 9.01557 | 1203.1 | 11483 | 4490 | 8.85462 | 1066.18 | 15381 | 9315 | 9.8821 | 1476.22 | 3398 | 1496 | 8.42037 | 967.76 |
| 15 | 10898 | 2369 | 7.76978 | 686.76 | 11646 | 3367 | 8.32026 | 850.24 | 3548 | 2372 | 9.36736 | 1394.52 | 15300 | 5859 | 8.86875 | 1069.2 |
| 16 | 3483 | 1763 | 8.95153 | 1101.26 | 4885 | 2176 | 8.78651 | 1036.7 | 6702 | 2376 | 8.3181 | 909.78 | 7145 | 3497 | 7.76274 | 1084.54 |
| 17 | 6644 | 3850 | 9.71854 | 1352.68 | 4694 | 3633 | 10.2724 | 1644.02 | 7044 | 3244 | 9.00293 | 1133.88 | 7791 | 3945 | 8.2994 | 1143 |
| 18 | 4582 | 2132 | 8.9605 | 1075.06 | 3780 | 2763 | 10.2501 | 1565.2 | 4961 | 2868 | 9.71107 | 1325.64 | 6248 | 3517 | 9.68846 | 1332.98 |
| 19 | 6678 | 1814 | 8.12013 | 747.56 | 5200 | 2661 | 9.34015 | 1194.98 | 14233 | 5996 | 9.16733 | 1154.4 | 10065 | 3987 | 8.53264 | 1046.12 |
| 20 | 6031 | 1375 | 7.78437 | 629.58 | 11232 | 5621 | 9.35836 | 1267.4 | 5838 | 4057 | 9.7997 | 1504.92 | 5945 | 3462 | 9.47831 | 1323.62 |
| 21 | 10080 | 1025 | 6.66398 | 384.42 | 3833 | 1807 | 9.13881 | 1076.78 | 6378 | 3780 | 9.54513 | 1348.46 | 4646 | 2203 | 8.41257 | 1074.18 |
| 22 | 4624 | 1660 | 8.41112 | 866.02 | 4958 | 3064 | 9.75755 | 1392.78 | 14529 | 8023 | 9.59986 | 1360.5 | 5934 | 3682 | 8.98784 | 1349.36 |
| 23 | 3590 | 1047 | 6.94969 | 662.92 | 5726 | 3403 | 9.80647 | 1368.3 | 3027 | 1879 | 9.54622 | 1309.4 | 3279 | 2528 | 10.2794 | 1613.34 |
| 24 | 5807 | 2441 | 8.77109 | 1016.14 | 4358 | 2070 | 9.14946 | 1100.16 | 16861 | 8847 | 9.62761 | 1336.74 | 6327 | 3752 | 9.7744 | 1379.04 |
| 25 | 7078 | 2625 | 8.29258 | 962.62 | 8021 | 3502 | 9.20984 | 1121.14 | 7483 | 3999 | 9.51972 | 1283.96 | 7523 | 3745 | 8.886 | 1181.78 |
| 26 | 4334 | 2223 | 9.12032 | 1149.4 | 6267 | 2814 | 8.23625 | 1077.36 | 8030 | 4014 | 9.50391 | 1243.36 | 5329 | 3379 | 9.46158 | 1410.64 |
| 27 | 8350 | 2871 | 8.78757 | 936.7 | 6519 | 2132 | 6.92213 | 796.28 | 5723 | 3513 | 9.557 | 1364.32 | 8190 | 4458 | 9.28773 | 1287.14 |
| 28 | 3365 | 1863 | 8.91662 | 1186.9 | 5680 | 3097 | 9.09341 | 1253 | 2383 | 1172 | 8.83008 | 1024.36 | 5721 | 2817 | 8.58799 | 1128.56 |
| 29 | 14512 | 4431 | 8.83628 | 955.94 | 7412 | 5249 | 10.0916 | 1566.06 | 6813 | 2599 | 7.76135 | 927.72 | 8972 | 4419 | 7.78379 | 1100.08 |
| 30 | 8332 | 3670 | 9.09865 | 1113.74 | 9002 | 4410 | 9.01626 | 1201.8 | 4424 | 2591 | 9.62214 | 1310.5 | 9751 | 4008 | 7.45059 | 966.72 |
| 31 | 4418 | 2029 | 9.10205 | 1065 | 6089 | 2320 | 7.8895 | 926.92 | 3298 | 1675 | 8.89997 | 1096.88 | 3242 | 1858 | 9.13036 | 1212.8 |
| 32 | 3043 | 1491 | 8.89069 | 1059.42 | 7326 | 3079 | 7.54848 | 1005.44 | 4089 | 2475 | 9.32446 | 1329.7 | 9445 | 5673 | 9.83695 | 1435.46 |
| 33 | 8532 | 3803 | 8.66723 | 1093.24 | 6846 | 1510 | 5.9281 | 579.42 | 5646 | 1752 | 6.98049 | 752.98 | 4945 | 2375 | 9.19564 | 1128.28 |
| 34 | 5170 | 2119 | 8.2388 | 953.18 | 5043 | 2811 | 9.5764 | 1280.64 | 4118 | 914 | 6.0315 | 515.72 | 15125 | 2756 | 7.98774 | 668 |
| 35 | 4430 | 2726 | 9.56502 | 1346.46 | 8658 | 514 | 3.7631 | 205.08 | 4079 | 2475 | 9.4205 | 1311.82 | 5583 | 3397 | 9.62212 | 1369.02 |
| 36 | 6080 | 2683 | 8.76857 | 1054.22 | 4501 | 2282 | 8.47307 | 1143.64 | 4267 | 1633 | 6.92658 | 874.22 | 6428 | 3742 | 8.73541 | 1301.32 |
| 37 | 5447 | 2206 | 7.79144 | 932.94 | 5146 | 3068 | 9.59455 | 1339.3 | 4440 | 1796 | 7.49758 | 924.02 | 4856 | 2622 | 9.39964 | 1232.94 |
| 38 | 7619 | 3941 | 9.11172 | 1248.12 | 7879 | 4642 | 9.5021 | 1362.92 | 5658 | 1786 | 8.27977 | 842.6 | 3138 | 2110 | 9.92049 | 1422.9 |
| 39 | 7487 | 4066 | 9.58663 | 1297.26 | 7679 | 4811 | 9.74067 | 1443.74 | 5100 | 992 | 5.59757 | 478.56 | 18976 | 11577 | 10.0716 | 1511.82 |
| 40 | 4178 | 2037 | 9.0123 | 1096.68 | 4044 | 1646 | 7.63996 | 908.12 | 4265 | 2405 | 9.46541 | 1256.26 | 8699 | 5149 | 9.87862 | 1401.6 |
| 41 | 5668 | 2827 | 9.63767 | 1228.42 | 8261 | 4036 | 9.40239 | 1239.5 | 7225 | 2908 | 8.62414 | 1012.72 | 3781 | 2514 | 9.66129 | 1409.62 |
| 42 | 7804 | 2761 | 8.52217 | 947.1 | 4342 | 2112 | 9.13799 | 1122.44 | 3388 | 1497 | 8.32155 | 973.34 | 4977 | 2478 | 9.10731 | 1144.66 |
| 43 | 4748 | 1681 | 8.15149 | 856.78 | 6386 | 3677 | 9.66081 | 1349.9 | 7812 | 3299 | 8.6741 | 1056.26 | 15247 | 4855 | 8.11919 | 964.94 |
| 44 | 6435 | 2952 | 8.88086 | 1119.74 | 8903 | 3793 | 9.09662 | 1114.32 | 8004 | 4441 | 9.48789 | 1335.08 | 6724 | 2759 | 8.58417 | 1009.04 |
| 45 | 3803 | 1904 | 8.57086 | 1083.76 | 2870 | 1411 | 9.11318 | 1064.1 | 8373 | 1629 | 6.10657 | 559.14 | 2991 | 2055 | 9.67834 | 1423.52 |
| 46 | 6294 | 2835 | 9.05956 | 1097.18 | 6869 | 4269 | 10.0081 | 1457.06 | 19675 | 5599 | 8.10396 | 888.6 | 4102 | 3113 | 9.9229 | 1591.02 |
| 47 | 7256 | 1617 | 8.10833 | 686.58 | 5090 | 2128 | 8.26437 | 967.68 | 7486 | 2795 | 8.41617 | 984.9 | 11552 | 3006 | 8.16469 | 795.48 |
| 48 | 9136 | 4147 | 9.06966 | 1147.38 | 6799 | 2886 | 8.40067 | 1069.16 | 7733 | 1505 | 7.20048 | 577.56 | 5180 | 3527 | 9.8874 | 1477.38 |
| 49 | 6849 | 1802 | 7.74354 | 736.98 | 7293 | 3499 | 9.06497 | 1157.88 | 5970 | 2481 | 8.72114 | 1021.1 | 5589 | 3280 | 9.51904 | 1334.1 |
| 50 | 4088 | 2632 | 9.88913 | 1411.22 | 8580 | 3227 | 8.88481 | 1037.18 | 8420 | 1953 | 7.88636 | 689.76 | 4950 | 3029 | 9.36604 | 1369.64 |
| 51 | 5831 | 2669 | 8.41818 | 1065.16 | 5103 | 2667 | 9.31184 | 1212.6 | 4240 | 1045 | 6.13304 | 591.86 | 15765 | 5322 | 8.83538 | 1021.74 |
| 52 | 11901 | 4220 | 8.12841 | 951.18 | 7506 | 4044 | 9.21853 | 1267.28 | 6140 | 2070 | 8.03339 | 834.48 | 4240 | 1987 | 7.99761 | 1012.36 |
| 53 | 9804 | 2275 | 8.05879 | 747.5 | 6140 | 3599 | 9.65359 | 1355.6 | 7056 | 2047 | 7.6566 | 765.88 | 4693 | 2857 | 8.78539 | 1304.26 |
| 54 | 12754 | 4219 | 8.89143 | 989.74 | 9107 | 4047 | 9.169 | 1138.92 | 7535 | 4501 | 9.50598 | 1386.44 | 5728 | 4548 | 10.3144 | 1678.3 |
| 55 | 12473 | 3902 | 7.91421 | 901.86 | 10535 | 2856 | 7.22414 | 756.38 | 5005 | 2798 | 9.73423 | 1298.58 | 4639 | 3093 | 9.93312 | 1456.12 |
| 56 | 7507 | 3187 | 9.01533 | 1066.86 | 3023 | 1538 | 8.58205 | 1092.62 | 5671 | 2295 | 7.18898 | 943.78 | 3422 | 1371 | 8.5501 | 903.1 |
| 57 | 4971 | 1869 | 8.26813 | 884.46 | 4701 | 2417 | 8.97844 | 1170.22 | 9384 | 1833 | 5.66095 | 560.3 | 4691 | 3399 | 9.80271 | 1533.2 |
| 58 | 3810 | 1482 | 8.07567 | 879.32 | 7576 | 2499 | 7.72925 | 825.1 | 8468 | 2987 | 7.37352 | 887.62 | 15020 | 4221 | 8.20874 | 865.98 |
| 59 | 4560 | 2696 | 9.70484 | 1319.9 | 4078 | 1218 | 6.86543 | 676.72 | 5448 | 1557 | 6.58684 | 670.3 | 5289 | 2570 | 8.21832 | 1061.62 |
| 60 | 6590 | 3830 | 9.5968 | 1351.72 | 5718 | 613 | 4.66936 | 293.12 | 5331 | 2908 | 9.3169 | 1241.94 | 3638 | 1302 | 8.1628 | 811.44 |
| 61 | 3793 | 1752 | 8.65391 | 1032.22 | 8308 | 4870 | 9.72381 | 1394.02 | 8566 | 3269 | 8.53942 | 966.4 | 5236 | 4103 | 10.4105 | 1678.8 |
| 62 | 2957 | 1201 | 8.13475 | 879.26 | 4750 | 1849 | 8.49857 | 919.48 | 6503 | 3735 | 9.62238 | 1333.38 | 6441 | 2775 | 8.33586 | 1007.56 |
| 63 | 5185 | 1557 | 7.80477 | 752.8 | 8910 | 1376 | 3.25932 | 391.02 | 5357 | 2577 | 9.31226 | 1154.06 | 7352 | 2724 | 7.29277 | 894.3 |
| 64 | 4991 | 1418 | 7.51816 | 703.84 | 6726 | 750 | 4.26666 | 323.98 | 4934 | 1986 | 7.65124 | 922.36 | 16443 | 7800 | 9.26387 | 1250.46 |
| 65 | 4038 | 1832 | 8.57015 | 1015.64 | 6417 | 3862 | 9.83269 | 1396.76 | 2353 | 1351 | 7.81601 | 1165.98 | 6895 | 4948 | 10.2002 | 1584.98 |
| 66 | 3418 | 1284 | 8.02243 | 841.2 | 5470 | 2324 | 8.08212 | 999.06 | 5913 | 3305 | 9.6577 | 1308.84 | 3267 | 2324 | 9.96019 | 1494.88 |
| 67 | 3950 | 1836 | 8.65839 | 1037.7 | 5185 | 3106 | 9.4312 | 1331.52 | 16826 | 7295 | 8.71514 | 1127.52 | 15807 | 3090 | 8.01617 | 712.26 |
| 68 | 7333 | 3416 | 8.94194 | 1124.98 | 7432 | 3435 | 8.38669 | 1096.88 | 5381 | 2715 | 8.93713 | 1153.08 | 8078 | 5963 | 10.1801 | 1623.28 |
| 69 | 4139 | 1642 | 8.73014 | 932.9 | 4556 | 2496 | 9.40391 | 1245.9 | 7831 | 2896 | 6.52404 | 857.68 | 3395 | 2501 | 10.1954 | 1561.94 |
| 70 | 5033 | 2681 | 9.30259 | 1216.86 | 5040 | 3195 | 9.59033 | 1389.78 | 3598 | 1907 | 9.30375 | 1179.4 | 4995 | 2221 | 8.75747 | 1022.7 |
| 71 | 5463 | 3007 | 9.36429 | 1250.88 | 8328 | 4762 | 9.80717 | 1383.32 | 14681 | 7509 | 9.46585 | 1330.24 | 3240 | 2114 | 9.71982 | 1380.06 |
| 72 | 6248 | 1591 | 6.9789 | 647.98 | 5204 | 2144 | 8.5661 | 976.3 | 7713 | 2912 | 7.14064 | 931.8 | 7425 | 2146 | 7.66011 | 791.76 |
| 73 | 8614 | 3300 | 8.02077 | 956.66 | 6335 | 1902 | 6.10481 | 719.82 | 8030 | 4014 | 9.48786 | 1238.04 | 6429 | 3441 | 9.5159 | 1275.4 |
| 74 | 7109 | 3469 | 9.04986 | 1159.84 | 3649 | 385 | 4.21797 | 257.38 | 3711 | 1802 | 9.14461 | 1095.8 | 2538 | 1272 | 9.08249 | 1059.52 |
| 75 | 14851 | 3732 | 5.85596 | 676.56 | 5917 | 2030 | 7.41752 | 821.94 | 2383 | 1172 | 8.83044 | 1022.38 | 8301 | 4059 | 9.44556 | 1218.1 |
| 76 | 6488 | 1493 | 6.86254 | 583.16 | 8221 | 2511 | 8.14605 | 844.52 | 3082 | 1660 | 9.18664 | 1158.46 | 4917 | 2811 | 8.33551 | 1227.22 |
| 77 | 7994 | 2627 | 7.32668 | 835.9 | 8427 | 1542 | 6.37793 | 532.62 | 7614 | 3941 | 9.47146 | 1251.74 | 5959 | 3067 | 9.56524 | 1235.66 |
| 78 | 6228 | 2767 | 9.09087 | 1090.42 | 8134 | 951 | 4.36268 | 339.38 | 7850 | 4484 | 9.71945 | 1372.52 | 7758 | 3735 | 8.58983 | 1130.6 |
| 79 | 8391 | 4450 | 9.76792 | 1317.84 | 5281 | 2304 | 9.2015 | 1078.94 | 4636 | 1711 | 8.02031 | 874.24 | 3458 | 1829 | 8.99532 | 1154.8 |
| 80 | 4066 | 1500 | 7.17774 | 831.74 | 6985 | 2439 | 8.53947 | 917.64 | 15342 | 3922 | 8.36987 | 848.02 | 6353 | 2587 | 8.5095 | 990.14 |
| 81 | 4822 | 1713 | 7.71111 | 838.64 | 3948 | 1640 | 8.1283 | 941.62 | 7205 | 1742 | 7.24325 | 658.68 | 6306 | 2250 | 6.8117 | 829.76 |
| 82 | 7492 | 2543 | 8.21629 | 893.02 | 8171 | 3978 | 9.14456 | 1184.92 | 3407 | 2049 | 9.40395 | 1292.64 | 4757 | 2670 | 9.73574 | 1288.42 |
| 83 | 6660 | 2089 | 6.66416 | 761.84 | 3861 | 1529 | 8.80923 | 933.74 | 3536 | 1989 | 9.49169 | 1236.9 | 2393 | 1548 | 9.71994 | 1330.88 |
| 84 | 8928 | 1917 | 4.27257 | 531.42 | 6033 | 1925 | 7.95123 | 801.86 | 3086 | 2278 | 10.1527 | 1544.22 | 3775 | 1405 | 7.6957 | 829.12 |
| 85 | 9855 | 1632 | 6.34063 | 523.44 | 5478 | 3733 | 9.77663 | 1491.2 | 13982 | 4014 | 6.97227 | 791.12 | 15326 | 7119 | 8.67201 | 1150.7 |
| 86 | 7248 | 2836 | 7.52193 | 938.86 | 8074 | 2984 | 8.31256 | 958.42 | 3179 | 1899 | 9.08137 | 1262.06 | 3829 | 2595 | 9.25686 | 1412.94 |
| 87 | 4167 | 1991 | 8.65446 | 1069.88 | 4642 | 1893 | 8.36985 | 959.06 | 14568 | 3954 | 8.11939 | 838.54 | 2557 | 1568 | 9.38381 | 1271.54 |
| 88 | 6729 | 3832 | 9.36796 | 1340.54 | 6026 | 3063 | 8.85876 | 1170.92 | 12361 | 7804 | 9.4915 | 1455.44 | 5339 | 3774 | 9.66969 | 1509.36 |
| 89 | 4883 | 2637 | 9.09654 | 1230.38 | 14882 | 3483 | 8.19746 | 789.2 | 4316 | 2892 | 9.98515 | 1467.84 | 3271 | 1805 | 9.33983 | 1192.7 |
| 90 | 10500 | 5145 | 9.58711 | 1284.34 | 4563 | 804 | 5.37402 | 433.64 | 9676 | 3794 | 8.57258 | 1018.74 | 11826 | 7022 | 9.12106 | 1376.46 |

Note: AC*, after cessation of probiotics administration
